# Role of Seed Therapy on Estrous and Non‐Estrous Cycle in Healthy Female Rats

**DOI:** 10.1002/fsn3.4692

**Published:** 2025-01-13

**Authors:** Iqra Majeed, Mahr Un Nisa, Muhammad Abdul Rahim, Mohamed Fawzy Ramadan, Fahad Al‐Asmari, Mohammed Alissa, Eliasse Zongo

**Affiliations:** ^1^ Department of Nutritional Sciences, Faculty of Medical Sciences Government College University Faisalabad Pakistan; ^2^ Department of Food Science & Nutrition, Faculty of Medicine and Allied Health Sciences Times Institute Multan Pakistan; ^3^ Department of Clinical Nutrition, Faculty of Applied Medical Sciences Umm Al‐Qura University Makkah Saudi Arabia; ^4^ Department of Food and Nutrition Sciences College of Agricultural and Food Sciences, King Faisal University Al‐Ahsa Saudi Arabia; ^5^ Department of Medical Laboratory College of Applied Medical Sciences, Prince Sattam bin Abdulaziz University Al‐Kharj Saudi Arabia; ^6^ Laboratory of Research and Teaching in Animal Health and Biotechnology Nazi Boni University Bobo‐Dioulasso Burkina Faso

**Keywords:** estrogen, fertility, hormones, oil seeds, phytoestrogens, reproduction

## Abstract

Seed cycling therapy (SCT) involves the consumption of specific seeds during the follicular and luteal phases of the menstrual cycle to help balance reproductive hormones. This study aimed to investigate the effects of SCT on healthy female Wistar albino rats to prevent hormonal imbalances. For SCT, a seed mixture (SM1) consisting of flax, pumpkin, and soybeans (estrogenic seeds) was administered at doses of 5.4, 4, 8, and 12 g per 100 g of diet during the non‐estrous phase. Another seed mixture (SM2) comprising sunflower, sesame, and chickpeas (also estrogenic) was given at doses of 3.12, 8, and 8 g per 100 g during the estrous phase. A total of 36 female Wistar albino rats were divided into four groups, each containing nine rats: Basal diet, seed cycling 1, seed cycling 2, and seed cycling 3 (SC_3_). All diets were isocaloric and iso‐nitrogenous. The results showed that body weight, feed intake, and water consumption were significantly decreased in the SC_3_ group (*p* < 0.05), with increased nutrient digestibility. The tested diets led to significant positive changes in levels of follicle‐stimulating hormone, luteinizing hormone, high‐density lipoproteins (HDL‐c), low‐density lipoproteins (LDL‐c), LDL‐c/HDL‐c ratio, aspartate aminotransferase, and alanine aminotransferase across both phases of the cycle. There was also a notable increase in estrogen, testosterone, prolactin, and insulin levels (*p* < 0.05). Ovarian histology results showed normal morphology in the SC_3_ group, suggesting that this dosage was the most effective. The findings indicate that further studies are warranted to explore the genetic mechanisms underlying phytoestrogen action during reproductive stages.

## Graphical abstract

Our study revealed the potential effects of seed cycling on reproductive profile, insulin, lipid, and liver enzymes in healthy rats. Higher intake of seeds showed an increase in estrogen, follicle‐stimulating hormone, and luteinizing hormone, while a decrease in testosterone, prolactin, and progesterone. It also significantly affects low‐density lipoprotein, high‐density lipoprotein, and insulin. High‐fiber crushed seeds showed significant weight loss by suppressing appetite. Graafian follicles showed maturation with the normal size of the antrum. The right ovary's granulosa cells and theca externa also showed significant results for the seed cycling 3 group. No toxicity was observed, indicating that safe levels of seed cycling were practiced in the study. The above dose is for a healthy person's intake and can be introduced into the daily diet of young girls in menarche to combat hormone imbalance during menstruation. Further studies are needed to examine the receptor activity behavior of phytoestrogens upon acute versus long‐term consumption.

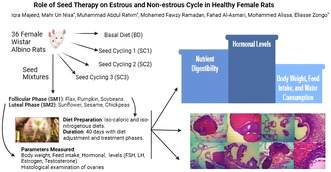

**Abbreviations**
AIN: American Institute of Nutrition
ALT: alanine aminotransferase
ANOVA: analysis of variance
AST: aspartate aminotransferase
BD: basal diet
ELISA: enzyme‐linked immunosorbent assay
ER: estrogen receptors
FCR: feed conversion ratio
FER: feed efficiency ratio
FSH: follicle‐stimulating hormone
HDL‐c: high‐density lipoprotein
LDL‐c: low‐density lipoprotein
LH: luteinizing hormone
LSD: least significant difference
MX: mineral mix
NFE: nitrogen‐free extract
PCOS: polycystic ovarian syndrome
SC_1_
: seed cycling 1
SC_2_
: seed cycling 2
SC_3_
: seed cycling 3
SCT: seed cycling therapy
SM1: seed mixture 1
SM2: seed mixture 2
TC: total cholesterol
TG: triglycerides
VX: vitamin mix

## 1 Introduction

Most oilseeds have been recognized as a good source of bioactive compounds, such as phytoestrogens, phenolic compounds, flavonoids, and tocopherol. Extensive research in nutrition and food engineering is needed to test the effective doses of oilseeds and their receptor‐binding behavior in different body metabolisms (Rahim et al. 2023).

Phytoestrogens, such as isoflavones and lignans are used as complimentary therapies in nutrition in that they act as alternatives to clomiphene, a nonsteroidal selective estrogen receptors (ER) modulator (Moini Jazani et al. 2019) due to the structural similarity with endogenous estrogen and having the potential to mimic and antagonize the action of endogenous estrogen by binding to estrogen α‐receptors and ß‐receptors. Traditional use of phytoestrogens is common to regulate the endogenous estrogen, which regulates the mechanism of the pituitary‐ovaries system and exerts a chemotactic effect on ovulation (Chen, Li, and Ou‐Yang 2022). Phytoestrogens can regulate abnormal hormonal status and are equally beneficial for producing hormones like insulin, melatonin reproductive hormones (estrogens and progesterone), luteinizing hormone (LH), and follicle‐stimulating hormone (FSH; Canivenc‐Lavier and Bennetau‐Pelissero 2023). The biological availability of phytoestrogens varies depending on the level of intake and the type of estrogen, route of entry, and administration. Phytoestrogens possess the properties of estrogen as well as antiestrogen capacity by binding with α‐ and ß‐receptors. This dual binding nature of phytoestrogen molecules to the ER makes them special for research. For example, soybeans containing phytoestrogen possess estrogenic and antiestrogenic effects in human cell lines (Cederroth, Zimmermann, and Nef 2012). Chickpeas lignin has shown the same behavior as their impact varies with the difference in doses to be administered (Canivenc‐Lavier and Bennetau‐Pelissero 2023). Phytoestrogens are equally beneficial for regulating menstruation in young girls, the problem of cysts in ovaries, and menopause in older women (Lobo 2019). All these problems are mainly due to hormonal imbalance and inflammation in the body caused by bad lifestyle, obesity, imbalanced diet, and psychological or physical stress (Borkar and Joshi 2023).

Phytoestrogens are plant‐derived compounds that can mimic or modify the activity of estrogen by binding to ERs (Lorand, Vigh, and Garai 2010). They play a crucial role in managing hormonal imbalances, particularly in conditions like estrogen deficiency or excess. Isoflavones, found in soybeans have estrogenic and antiestrogenic properties (Tripathi et al. 2013), while lignans in flaxseeds influence estrogen metabolism (Brooks et al. 2004). Domínguez‐López et al. (2020) found that dietary phytoestrogens can regulate hormonal levels, impact reproductive health, bone density, and cardiovascular health, potentially reducing conditions like breast cancer and osteoporosis. Furthermore, Swathi Krishna, Kuriakose, and Lakshmi (2022) found phytoestrogens positively impact reproductive organ health, regulating ovarian function, promoting follicular development, and mitigating estrogen imbalances, which can lead to reproductive disorders.

Many pharmaceutical and nutritional therapies have been used for hormonal balance. The main focus is to balance the levels of insulin and androgen hormones, which play a vital role in inflammation and estrogen synthesis and balance (Cederroth, Zimmermann, and Nef 2012). The seeds traditionally regulate women's hormones by providing the nutraceutical compounds in the functional foods they need at various reproductive cycle stages. Seed cycling therapy (SCT) is now trending to treat polycystic ovarian syndrome (PCOS) and balance reproductive hormones (Rasheed et al. 2023). Without any scientific evidence, many nutritionists recommend seed cycling to improve menstruation. However, few studies are available that describe seed cycling, as work in 2019 suggests that using these seeds helps prolong the follicular phase, the phase of follicle formation inside the ovaries, by lowering the FSH, LH, and estrogen concentration (Lobo 2019). SCT involves flax, pumpkin, sesame, and sunflower seeds. In this study, different proportions of oil seeds are used for the follicular (flax, soybeans, and pumpkin) and luteal stage (sunflower, chickpeas, and sesame) of female rats during the menstrual cycle due to their phytoestrogen properties.

## 2 Materials and Methods

### 2.1 Study Setting and Ethical Approval

The experimental procedure was approved by the Secretary and Chairperson of the Ethics Review Committee of Government College University, Approval No: GCUF/ERC/86, Date: 11/04/2022. 65‐day‐old female *Wister Albino* rats weighing 160 ± 10 g were taken from Lahore, Punjab, Pakistan. Female rats were kept and treated according to the principles of laboratory animal care (National Institutes of Health) and guidelines for the management of laboratory animals (National Research Council 2010; Ghasemi, Jeddi, and Kashfi 2021). They were kept at a relatively humid condition of 45%–55% with a temperature of 25°C ± 2°C under a 12‐h light and dark cycle. They were housed in clean metabolic cages for feces collection purposes. Animals had free access to pellet food and water ad libitum with an acclimatization period of 2 weeks before the experiment. The total duration of the trial was 40 days, out of which 15 days were for diet adjustment and 25 days were for seed mixtures (SM1 and SM2) intake and the estrus cycle study.

### 2.2 Study Design and Estrus Cycle Determination

**FIGURE 1:**
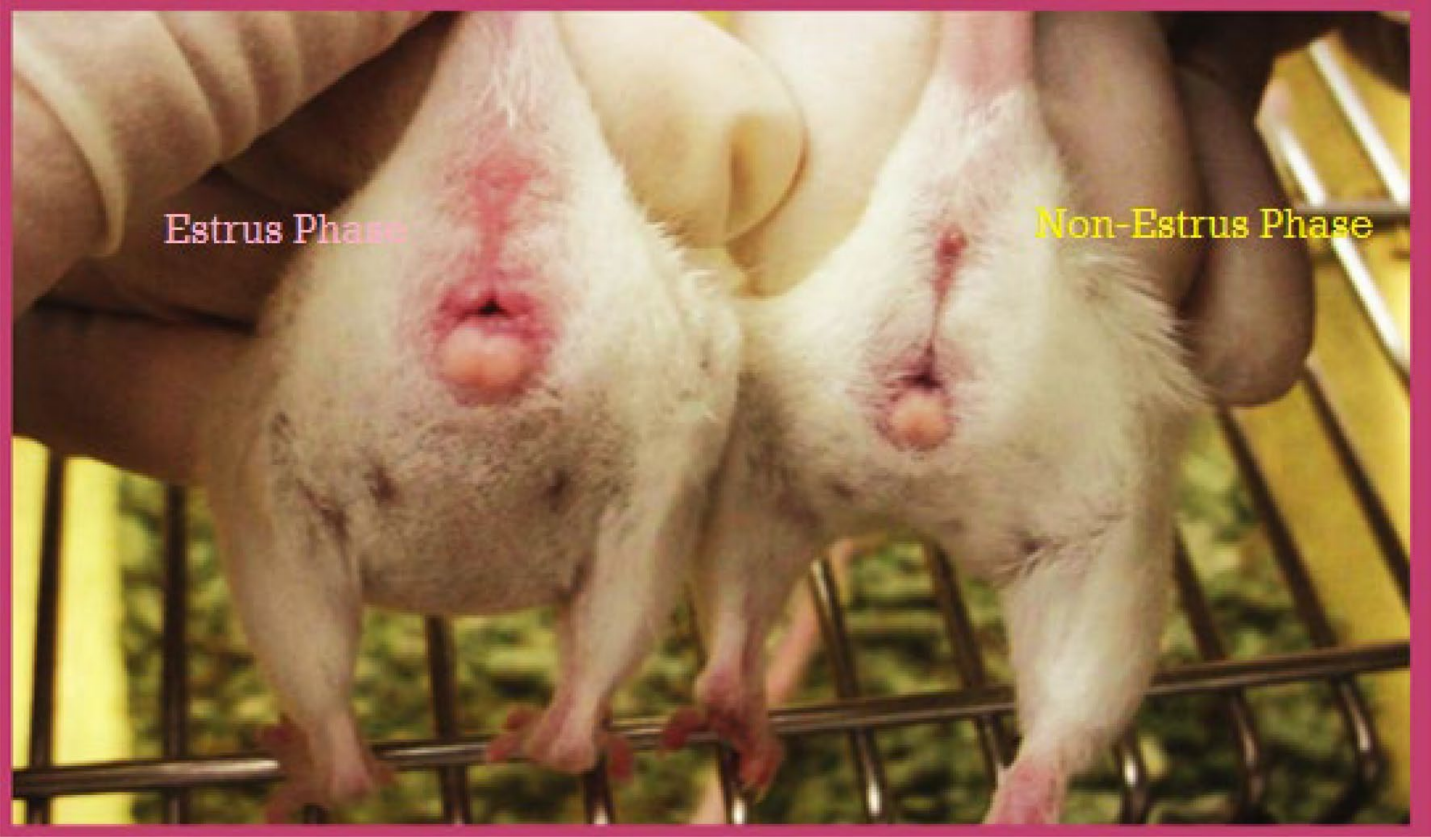
Estrus and non‐estrus phases of healthy female rats indicated by different colors.

Based on physical observation, the estrus cycle phases of each rat were determined. Rats with the estrus phase had swollen, moist, and open appearance of the vaginal opening, while in the non‐estrus phase, the vaginal opening was unswollen and closed (Marcondes, Bianchi, and Tanno 2002). Rats were then divided into corresponding groups based on cycle phases at the trial's initiation. Thirty‐six rats were arranged in four groups, each group containing nine rats. At the start of the trial, the basal diet (BD) group was in the non‐estrous phase, seed cycling 1 (SC_1_) was in the estrous phase, seed cycling 2 (SC_2_), and seed cycling 3 (SC_3_) groups were in the non‐estrous phase (Figure 1).

### 2.3 Composition of the Diet

*Isocaloric* and *iso‐nitrogenous* diets are shown in Tables 1 and 2. The standard diet comprises all the required micronutrients (minerals and vitamins) as per the American Institute of Nutrition (AIN‐93) guideline for rats, and intake was recorded daily (National Research Council 1995). Each rat's body weight was noted weekly using a digital electronic weighing balance (SF‐400).

**TABLE 1:** Feed ingredients of SM1 for the follicular phase of the estrous cycle of rats (with treatment seeds and other ingredients).

Ingredients (g/100 g)	BD (g)	SC_1_ (g)	SC_2_ (g)	SC_3_ (g)
Dextrose	25	25	25	25
Corn starch	13.25	10.81	7.75	4.75
Crude oil	7	4	4.5	3.5
Canola meal	50	50	50	50
AIN‐93‐VX (vitamin mix)	1	1	1	1
AIN‐93G‐MX (mineral mix)	3.5	3.5	3.5	3.5
Choline bitartrate	0.25	0.25	0.25	0.25
SM1	0	5.44	8	12

**TABLE 2:** Feed ingredients of SM2 for the luteal phase of the estrous cycle of rats (with treatment seeds and other ingredients).

Ingredients (g/100 g)	BD (g)	SC_1_ (g)	SC_2_ (g)	SC_3_ (g)
Dextrose	25	25	25	25
Corn starch	13.25	14.13	10.25	10.25
Crude oil	7	3	2	2
Canola meal	50	50	50	50
AIN‐93‐VX (vitamin mix)	1	1	1	1
AIN‐93G‐MX (mineral mix)	3.5	3.5	3.5	3.5
Choline bitartrate	0.25	0.25	0.25	0.25
SM2	0	3.12	8	12

*Note:* SM1 (flax, soybeans, pumpkin), BD, basal diet without seeds, SC_1_: seed cycling 1 (flax: 0.72, soy: 4, pumpkin: 0.7, total: 5.44 g), SC_2_: seed cycling 2 (flax: 2, soy: 4, pumpkin: 2, total: 8 g), SC_3_: seed cycling 3 (flax: 4, soy: 6, pumpkin: 2, total: 12 g). SM2 (sunflower, chickpeas, sesame), SC_1_ (sunflower: 1.28, chickpea: 0.84, sesame: 1, total: 3.12 g), SC_2_ (sunflower: 4, chick: 2, sesame: 2, total: 8 g), SC_3_ (sunflower: 4, chickpea: 2, sesame: 2, total: 12 g), AIN, American Institute of Nutrition; MX, mineral mix; NFE, nitrogen‐free extract; VX, vitamin mix.

### 2.4 Diet Preparation

Different levels of seed mixtures were adjusted in a 100 g BD. All diet ingredients were added as per diet plans for each study group. Diet was measured carefully by using a digital electronic weighing balance. After measuring, all ingredients were appropriately mixed in a bucket. Soft pallets of different sizes were prepared with water and allowed to dry for at least 24 h. Before serving, the diet was measured on a weighing scale and offered according to Nutrient Requirements of Laboratory Animals nutrition guidelines (National Research Council 1995). All diets were provided to the treatment groups based on the estrus cycle stage. Because the length of the estrous cycle of rats lasts for 4 days, the SM1 diet was given for 2 days in the non‐estrous phase, while the SM2 diet was shown in the next 2 days with the estrus phase (Marcondes, Bianchi, and Tanno 2002). Feed was offered in the form of pallets.

### 2.5 Determination of Growth Performance and Nutrient Digestibility

Feed for each group was measured and recorded as daily intake throughout the trial to observe the rats' feed acceptance and intake behavior. In the last 7 days of the trial, feces were collected, weighed, and stored at −20°C for nutrient digestibility. Proximate analysis of feed and feces was performed using the official analysis methods by the Association of Analysis Chemists 2006 to check the nutrient digestibility. All the parameters of growth performance, including feed intake, feed conversion ratio (FCR), feed efficiency ratio (FER), and nutrient digestibility, were determined according to the following Equations ((1), (2), (3)) (AOAC 2006).

(1) $$\mathrm{FCR}=\frac{\text{Feed intake}\;\left(g\right)}{\text{Body weight}\;\left(g\right)}$$

(2) $$\mathrm{FER}=\frac{\text{Body weight}\;\left(g\right)}{\text{Feed intake}\;\left(g\right)}$$

(3) $$\text{Digestibility}\;\left(\%\right)=\frac{\text{Nutrient intake}-\text{Nutrient in feces}}{\text{Nutrient intake}}\times 100$$

### 2.6 Animal Sacrifice and Sample Collection

After the last dose administration, the animals were fasted overnight for 12 h. Two rats from three replicates were first temporarily anesthetized by chloroform (Sigma Aldrich, St. Louis, MO, USA) and given an intraperitoneal injection with the drug (xylazine with ketamine, 2:1 ratio) to increase the anesthesia time. Before beginning sample collection, test tubes were labeled with the groups' names. Samples were taken after anesthetizing the rats in a 5% chloroform solution. Blood samples were collected by cutting the neck area and exposing the jugular vein with a sharp scalpel blade. Approximately 5 cc of blood was drawn out, and placed in ethylene‐di‐amine‐tetra‐acetate (EDTA) (Yang et al. 2018). Serum was obtained by centrifugation at 500 rpm for 20 min and stored at −20°C for the biochemical analysis, after which the rats were decapitated, and the right and left ovaries were segregated and preserved in 10% formalin solution for histological examination (Donczo and Guttman 2018).

### 2.7 Biochemical Profile

Serum levels of total cholesterol (TC), serum low‐density lipoproteins (LDL‐c), and triglycerides (TG) were spectrometrically measured (BIOLAB‐310; Biobase, Jinan, China), after enzymatic hydrolysis and oxidation, with enzymatic calorimetric method (Ihedioha, Noel‐Uneke, and Ihedioha 2013). A high‐density lipoproteins (HDL‐C) direct FS kit determined an HDL‐c level enzymatically (DiaSys Diagnostic System GmbH, Germany). Liver enzyme assay, alanine aminotransferase (ALT), and aspartate aminotransferase (AST) were carried out enzymatically by using a Bioactive's ALT (GPT) SR kit (catalogue # 10498‐99‐93‐183) and a commercially available liquiform technique kit (Crescent Diagnostic kit, Jeddah, catalogue #15204C for AST).

### 2.8 Hormonal Profile

LH, FSH, progesterone, testosterone, prolactin, and estrogen were analyzed using enzyme‐linked immunosorbent assay (ELISA) test kit (Biocheck Inc. Foster City, CA 94404 USA). Serum insulin level was measured using a kit (Calbiotech; Catalogue No. IN374S; El Cajon, CA, USA). ELISA microwells for reproductive profile and insulin purchased from Scientific Store Faisalabad, Pakistan.

### 2.9 Histopathological Studies

Ovarian tissues were fixed in a 10% neutral buffered formalin solution immediately after excising them. Tissue sections were cut to a thickness of 5 μm for histopathological examination. After 3 days of fixation, the samples were removed and rinsed to remove the fixative. Tissue samples were then dehydrated with 95% ethanol. After dehydration, Xylene was used as a clearing agent, and melted liquid paraffin was used as an embedding medium. The tissues were infiltrated in it for tissue blockage. Slides were observed under a light microscope XSZ 107BN optical (Zenith Lab IBD, China) with an attached OptikamB1 digital camera (Optika Microscopes, Italy). To see primary and secondary follicle development, all images were acquired digitally using Optika IS view imaging software (Optika Microscopes, Italy) at 40× and 100× magnifications. This procedure of histopathology was based on the study by Okafor, Nnamah, and Nnaka (2021).

### 2.10 Statistical Analysis

All the data were measured for analytical means and standard deviation. Data analysis used Statistics 8.1 (analytical software, Tallahassee, FL, USA). All the data were tested using one‐way analysis of variance (ANOVA), and then the least significant difference (LSD) was applied post hoc. A complete randomized design was assigned to randomize each treatment. Results and values were considered as significant at *p* < 0.05.

## 3 Results

### 3.1 Effect on Feed Intake, Body Weight, Water Intake, and Its Efficiency Parameters

After 3 weeks of trial, feed intake significantly decreased in treatment groups compared to the BD group. Significant decreases of SC_1_ (17.37 ± 0.7 g) and (15.47 ± 0.5 g) were observed in the groups SC_2_ and SC_2_ as compared to the SC_3_ (15.71 ± 0.5 g). SC_3_ had shown a mild decrease in feed intake (Tables 3 and 4). FCR significantly improved in SC3 groups compared to the other treatment groups (SC_1_, SC_2_, and BD), as shown in Table 3. In other groups, FCR significantly decreased due to the negative impact of treatment diets on feed intake and body weight. A similar trend was seen in the values of FER among the treatment groups. FCR and FER tell us about the impact of feed utilization on body weight.

**TABLE 3:** Effects of dietary supplementation of seed mixtures on growth performance and FCR and FER.

Groups	BD	SC_1_	SC_2_	SC_3_	*p*
Total feed intake in 3 weeks per rat (g/day)	18.78 ± 0.9^a^	17.37 ± 0.7^b^	15.47 ± 0.5^c^	15.71 ± 0.5^c^	0.02^*^
Total weight gain in 3 weeks per rat (g/day)	0.66 ± 0.03^a^	0.73 ± 0.04^a^	1.39 ± 0.6^a^	0.39 ± 0.1^a^	0.22^NS^
Average weight gain	19.81 ± 0.9^c^	22.04 ± 1.1^b^	41.86 ± 2.1^a^	11.85 ± 0.5^d^	0.03^*^
FCR	28.45 ± 1.4^b^	23.79 ± 1.1^c^	11.12 ± 0.5^d^	40.28 ± 2.1^a^	0.04^*^
FER	3.51 ± 0.1^c^	4.2 ± 0.2^b^	2.4 ± 0.4^a^	8.9 ± 0.6^d^	0.02^*^
*Total water intake in 3 weeks (mL)*	*93.13 ± 0.15* ^*a*^	*90.23 ± 0.04* ^*a*^	*92.9 ± 0.09* ^*a*^	*91.46 ± 0.07* ^*b*^	*0.15^NS^*

*Note:* (a to d)—Means with different superscripts differ significance effect (*p* < 0.05). Data were analyzed using one‐way ANOVA, followed by multiple comparisons using LSD, and data were considered significant at *p* ≤ 0.05.

Abbreviations: BD, basal diet; FCR, feed conversion ratio; FER, feed efficiency ratio; SC_1_, seed cycling 1; SC_2_, seed cycling 2 group; SC_3_, seed cycling 3 group.

*p ≤ 0.05 means significant and *p* > 0.05 means non‐significant (NS).

**TABLE 4:** Effect of different levels of SM1 and SM2 on body weight of healthy female rats.

Body weight (g)	BD	SC_1_	SC_2_	SC_3_	*p*
Initial body weight (before treatment)	114.66 ± 5.7^b^	108.55 ± 6^c^	129 ± 4^a^	106.66 ± 5^d^	0.01^*^
Weight on day 10	102.33 ± 3.1^b^	94.55 ± 2.5^d^	98.88 ± 2.9^c^	103.22 ± 3.1 ^a^	0.03^*^
Weight on day 20	95.22 ± 2.6^b^	86.77 ± 2^c^	88 ± 1.5^c^	97.44 ± 1.7^a^	0.04^*^
Average body weight gain (After treatment)	87.33 ± 2.1^a^	78.22 ± 1.3^c^	74.55 ± 1.2^d^	83.77 ± 1.5^b^	0.02^*^

*Note:* (a to c)—Means with different superscripts differ in significance effect (*p* < 0.05). Data were analyzed using one‐way ANOVA, followed by multiple comparisons using LSD, and data were considered significant at *p* < 0.05.

Abbreviations: BD, basal diet; SC_1_, seed cycling 1; SC_2_, seed cycling 2 group; SC_3_, seed cycling 3 group; SM1, seed mixture 1; SM2, seed mixture 2.

*p ≤ 0.05 means significant and *p* > 0.05 means non‐significant (NS).

### 3.2 Nutrient Digestibility

The effect of diet on nutrient digestibility and weight changes is shown in Table 5. The digestibility analysis gave significant results in all treatment groups. The highest dry matter, crude fiber, ether extract, crude protein, and ash digestibility was observed in SC_3_ level as compared with BD and other treatment groups, while a reduction trend was observed in ash, dry matter, crude protein, and NFE of SC_1_ and SC_2_ as compared to BD and SC_3_. The seed mixtures showed a reduction (*p* < 0.05) trend in body weight gain of SC_1_, SC_2_, and SC_3_ compared to BD. However, a significant reduction was noticed in SC_2_ as compared to BD.

**TABLE 5:** Effects of dietary supplementation of seed mixtures on nutrient digestibility.

Parameter (%)	BD	SC_1_	SC_2_	SC_3_	*p*
Dry matter	78.94 ± 2.3^a^	73.29 ± 2.2^b^	70.69 ± 1.9^b^	79.31 ± 2.3^a^	0.03^*^
Crude protein	67.27 ± 1.6^a^	60 ± 1.2^b^	56.2 ± 1.1^c^	67 ± 1.5^a^	0.03^*^
Ether extract	40 ± 0.7^c^	40 ± 0.2^c^	48 ± 0.8^b^	55 ± 1.1^a^	0.02^*^
Crude fiber	56.36 ± 1.1^b^	39 ± 0.9^d^	43 ± 0.7^c^	71.72 ± 2^a^	0.04^*^
Ash	52.96 ± 0.9^b^	42.10 ± 0.7^c^	33.33 ± 0.4^d^	55.79 ± 1^a^	0.01^*^
NFE	52.26 ± 0.9^b^	43.10 ± 0.7^c^	36.16 ± 0.6^d^	56.72 ± 1.1^a^	0.04^*^

*Note:* (a to d)—Means with different superscripts differ significance effect (*p* < 0.05). Data were analyzed using one‐way ANOVA, followed by multiple comparisons using LSD, and data were considered significant at *p* < 0.05.

Abbreviations: BD, basal diet; NFE, nitrogen free extract; SC_1_, seed cycling 1; SC_2_, seed cycling 2 group; SC_3_, seed cycling 3 group.

*p < 0.05 means significant and *p* > 0.05 means non‐significant (NS).

### 3.3 Hormonal Profile

The phytoestrogenic diet exerted a positive impact on hormone levels by increasing estrogen (pg/mL), progesterone, testosterone, and prolactin levels with a reduction trend in FSH, LH, LH/FSH ratio, and serum fasting insulin, as shown in Table 6. Significantly lowest concentration of estrogen was observed in SC_1_ compared to other treatments, while it was highest in SC3 rats compared to SC_2_ and BD in the non‐estrous phase. Significant (*p* < 0.05) highest progesterone level in rats was observed in SC_2_ compared to SC_3_ and BD. There was a significant (*p <* 0.05) synergic decrease of progesterone with estrogen in SC_1_ with estrous phase but higher than BD because of phase difference (non‐estrus). Testosterone and prolactin showed a significant (*p <* 0.05) decrease in SC_3_ compared with other treatments. It was observed that serum insulin levels, FSH, and LH reduced significantly (*p <* 0.05) in all rats fed with SC_1_, SC_2_, and SC_3_ diets. However, a significant decrease was shown in SC_2_ when compared with SC_1_ and SC_3_, while the LH/FSH ratio remained lower than 2 at all levels.

**TABLE 6:** Impact of SM1 and SM2 levels on hormonal profile in healthy rats.

Parameter	BD	SC_1_	SC_2_	SC_3_	*p*
Estrogen (pg/mL)	2.11 ± 0.02^d^	3.43 ± 0.09^c^	4.51 ± 0.06^b^	6.67 ± 0.13^a^	0.04^*^
Progesterone (ng/mL)	0.24 ± 0.02^d^	0.32 ± 0.01^c^	0.71 ± 0.03^a^	0.57 ± 0.06^b^	0.01^*^
FSH (mlU/mL)	0.75 ± 0.05^a^	0.39 ± 0.05^c^	0.30 ± 0.08^c^	0.47 ± 0.01^b^	0.04^*^
LH (mlU/mL)	0.78 ± 0.01^a^	0.52 ± 0.03^b^	0.39 ± 2.0^c^	0.55 ± 0.03^b^	0.02^*^
LH: FSH	1.04	1.3	1.3	1.17	
Testosterone (ng/mL)	0.85 ± 0.03^c^	1.68 ± 0.07^b^	2.31 ± 0.18^a^	1.62 ± 0.13^b^	0.01^*^
Prolactin (ng/mL)	2.4 ± 0.11^c^	3.47 ± 0.21^b^	4.83 ± 0.13^a^	4.35 ± 0.14^a^	0.03*
Insulin (μU/mL)	4.68 ± 0.13^b^	3.14 ± 0.34^c^	3.63 ± 0.4^c^	5.77 ± 0.19^a^	0.04^*^

*Note:* (a to d)—Means with different superscripts differ significance effect (*p* < 0.05). Data were analyzed using one‐way ANOVA, followed by multiple comparisons using LSD, and data were considered significant at *p* < 0.05.

Abbreviations: BD, basal diet with non‐estrous phase; FSH, follicle‐stimulating hormone; LH, luteinizing hormone; SC_1_, seed cycling 1; SC_2_, seed cycling 2 group; SC_3_, seed cycling 3 group in non‐estrous phase; SM1, seed mixture 1; SM2, seed mixture 2.

*p < 0.05 means significant and *p* > 0.05 means non‐significant (NS).

### 3.4 Biochemical Profile

The serum biochemical profile of BD after the intake of seed mixtures in SC_1_, SC_2_, and SC_3_ levels of healthy female rats is shown in Table 7. A significant reduction (*p <* 0.05) was observed in LDL‐C levels. Maximum decrease was observed in SC_3_ compared to BD and other treatment groups, while significant (*p* < 0.05) improvement in HDL‐c levels was observed. All treatments' TG was insignificant (*p* > 0.05) with no improvement in TC levels compared with BD. ALT showed highly significant results (*p* < 0.05) among all treatment groups. Compared with BD and reference range, the SC_2_ group gave the best results by significantly (*p <* 0.05) reducing ALT level. AST level showed significantly increased results (*p* < 0.05) for SC_1_ and SC_3_ with nonsignificant (*p* > 0.05) results for SC_2_ as compared to BD.

**TABLE 7:** Impact of different levels of SM1 and SM2 on biochemical profile in healthy female rats.

Parameter	BD	SC_1_	SC_2_	SC_3_	*p*
HDL‐c (mg/dL)	16 ± 1^b^	16.66 ± 0.5^b^	13.33 ± 1.5^c^	19 ± 1^a^	0.01^*^
LDL‐c (mg/dL)	105 ± 5^a^	102 ± 4^b^	94 ± 3^c^	75.33 ± 2.5^d^	0.02^*^
TG (mg/dL)	105.6 ± 5.0^a^	110 ± 12^a^	108 ± 6.0^a^	110.33 ± 13^a^	0.88^NS^
TC (mg/dL)	80.66 ± 2.5^c^	90 ± 3^b^	78.33 ± 2.5^d^	98.66 ± 3.5^a^	0.03^*^
ALT (U/l)	12 ± 1^c^	20 ± 0.5^a^	9 ± 0.9 ^d^	18 ± 1 ^b^	0.04^*^
AST (U/l)	12 ± 1^b^	21.33 ± 0.5^a^	13 ± 1 ^b^	23 ± 1.2 ^a^	0.01^*^

*Note:* (a to d)—Means with different superscripts differ significance effect (*p* < 0.05). Data were analyzed using one‐way ANOVA, followed by multiple comparisons using LSD, and data were considered significant at *p* < 0.05.

Abbreviations: ALT, alanine aminotransferase; AST, aspartate transferase enzyme; BD, basal diet; HDL‐c, high‐density lipoproteins; LDL‐c, low‐density lipoproteins; SC_1_, seed cycling 1; SC_2_, seed cycling 2 group; SC_3_, seed cycling 3 group; SM1, seed mixture 1; SM2, seed mixture 2; TC, total cholesterol; TG, triglycerides.

*p < 0.05 means significant and *p* > 0.05 means non‐significant (NS).

### 3.5 Histopathology

**FIGURE 2:**
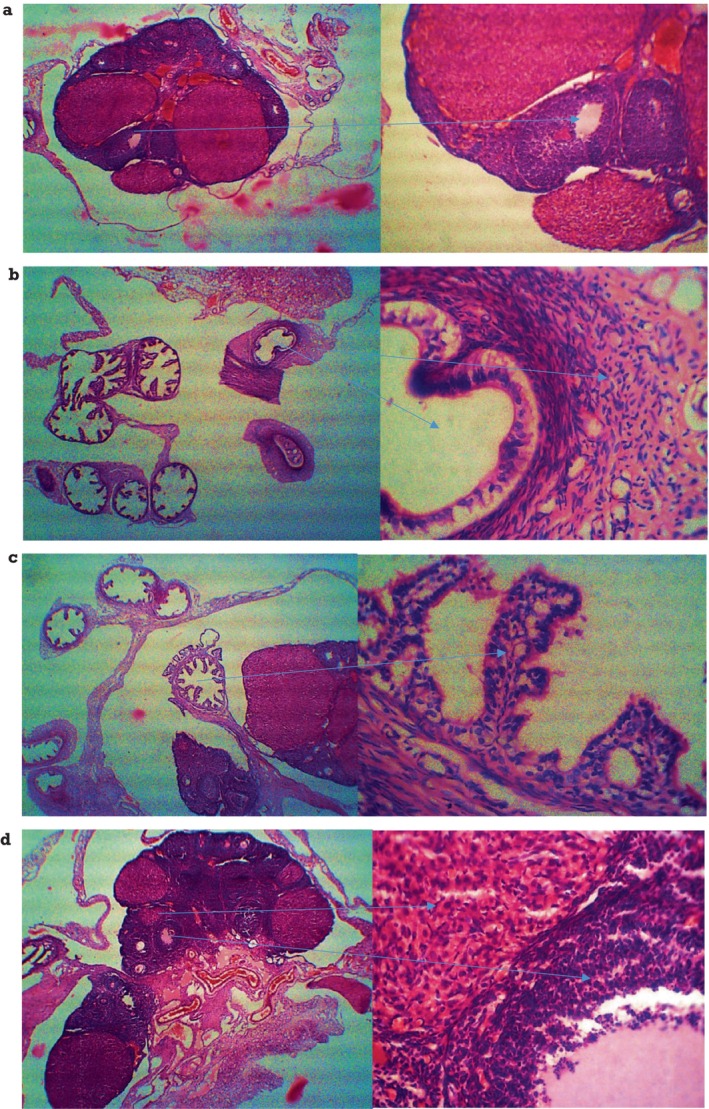
Ovarian histopathological examination of healthy female rats. Histological sections were stained with hematoxylin and eosin. (a) In BD groups (a H&E× 100), various stages of follicles were developed normally with aggregation of granulosa cells. (b–d) In treatment groups (b H&E× 100), the chance of formation of follicular cysts is decreased with less degrading and thin layers of granulosa cells. After treatment of different levels of seed cycling therapy, a few follicles with various developmental stages were observed in groups treated with SC_1_, SC_2_ and SC_3_ diets (c–e: H&E × 100). CF, cystic follicles; CL, corpus luteum; GC, granulosa cells; O, corpus luteum.

Figure 2a. served as a control group and represented the histological section of the ovary of the rat administered with BD throughout the experiment. The micrograph shows a normal ovary with normal follicular development and corpus lutea, indicating ovulation with the normal size of the antrum and granular cells and normal thickening of theca interna and theca externa. The photography was taken at 40× and 100× magnifications. Figure 2b shows the histology of the ovary of treatment group seed cycling 1. This section shows characteristics of depletion of granulosa cells, regressed cumulous oophorous, and decreased thickness of zona pellucida. Figure 2c shows the right and left ovaries of rats from treatment group 2. The histology sections from this group showed multiple follicular cysts with blood vessel growth. Figure 2d showed the micrographs of the right and left ovaries with the normal size of the antrum, normal structures of cumulous oophorous, zona pellucida, granulosa cells, and thickness of theca interna and theca externa at 40× and 100× magnifications.

## 4 Discussion

Many synthetic and conventional medications are used to treat different diseases. However, they all have different side effects. Thus, there is a growing global interest in traditional and organic medicinal plants. Due to their diverse therapeutic properties, various herbal plants have been used to prevent the occurrence and incidence of various disorders. Many seed oils contain important active compounds such as phytoestrogen, phenolic compounds, flavonoids, and tocopherol. These compounds combat different ailments and contain aberrant effects on reproductive health (Morya et al. 2022; Rahim et al. 2023). During the experimental period, body weight was significantly decreased among the treatment groups compared to the normal control group. This might be attributed to the decrease in digestibility and hypolipidemic activity of seeds. This stance increases the activity of lipid enzymes, subsequently increasing the lipid metabolism in the liver. Parallel to this, a substantial decrease in water intake observed might be linked to the lower feed intake (Preedy and Watson 2020). There was a slight increase in feed intake, body weight, and digestibility in the SC_3_ group, but an overall nonsignificant trend was observed in the normal control group compared to the treatment groups, as shown in Tables 3 and 4. Water intake decreased due to the decrease in feed intake and digestibility of nutrients, as shown in Table 3. Findings from this study showed a significant (*p* < 0.05) increase in nutrient digestibility of dry matter, crude fiber, ether extract, crude protein, and ash digestibility (Table 5). The digestibility trend for crude protein, ether extract, crude fiber, ash, and NFE (nitrogen‐free extract) (carbohydrates) seems better in SC_3_ and decreased in the other treatment groups compared to normal control groups. This mechanism has been linked to lower feed intake and antiquality (phytates) compounds in the seeds and feed. These compounds form complexes in the intestine, resulting in a decrease in nutrient absorption. Dry matter digestibility was nonsignificant (*p* > 0.05) in the treatment groups compared to the normal control group. Improved nutrient digestibility in the SC_3_ groups was positively linked with improved feed and fiber intake. Fiber intake facilitates the commensal microbes in the intestine, resulting in better assimilation and absorption of nutrients in the intestine (Vlaicu et al. 2019; Manzoor et al. 2020). Another study endorses this study's findings. A decrease in the digestibility of nutrients might also linked with a decrease in the feed intake and less release of the ghrelin hormone responsible for the appetite. It may attributed to the antiquality compounds in the treatment diets, which may halt the absorption of valuable nutrients (Fagbenro, Adeparusi, and Jimoh 2013). According to Vlaicu et al. (2019), the nutrient digestibility increased with increased seed quantity of flax seeds and pumpkin waste with grape seed meals in pigs. Flax seeds contain omega‐3 fatty acids, essential oils that have an anti‐inflammatory effect. In another study, Fagbenro, Adeparusi, and Jimoh (2013) reported that the apparent nutrient digestibility coefficients significantly (*p* < 0.05) improved with 15%, 30%, and 45% sunflower and sesame seeds replacement with soybean meal in clariid catfish. The reduced fiber digestibility helps to improve feed intake, so the effect of feed intake was nonsignificant (*p* > 0.05) in the present study. A reduction in weight in treatments may be due to the weight‐loss properties of seeds, as shown in Table 5. This was also co‐related with previous study results that phytate in seeds interferes with feed digestion and reduces weight (Cheng and Hardy 2004). FCR and FER are important parameters in animal trials, especially in agriculture and husbandry. This provides valuable insights into the impact of the feed on the body. FCR indicates how nutrients from the feed become part of the body. In this study, FCR did not improve due to decreased body fat and body weight, as shown in Table 3. This might be linked to the lipid‐lowering effect of the seed‐cycling compounds in the diet (Komal et al. 2020).

The hormonal levels (estrogen, progesterone, LH, FSH, testosterone, prolactin, and insulin) were determined in this study. The highest concentration of estrogen might be due to higher seed doses. Previous literature observed that flaxseed intake or enriched diet with flaxseed powder significantly reduced the LH values (Farzana et al. 2015; Nowak et al. 2007). LH is important in androgen production, and theca cells are feasible to synthesize the androgen in higher concentrations. Essential fatty acids in seeds, including omega‐3 fatty acids, significantly decreased the androgen production in the body (Sturgeon et al. 2008). Patients with PCOS suffer from various metabolic conditions such as inflammation, insulin resistance, and free radicals exposure (Zheng and Li 2016). Various seeds contain essential fatty acids that show an aberrant effect on the body's fats, insulin sensitivity, and inflammatory markers, including tumor necrosis factor and interleukine‐6. They also help the production and release of adiponectin (Monk et al. 2014). Phytoestrogens are equally beneficial in reproductive age to balance estrogen and in menopause to restore estrogen losses, as explained in a study by Haggans et al. (1999) that 5–10 g of daily flax seeds consumption exerts chemo‐protective effects against breast cancer and maintaining normal estrogen status in women. This might be due to the body's balance in antioxidant status. Moreover, HaiRong et al. (2013) explained that chickpea consumption helps to restore endogenous estrogen and improve estrogen deficiency‐based osteoporosis. Normal levels of FSH and LH are crucial for the regulation of the reproductive cycle as an imbalance in these levels can lead to many complications of ovarian diseases like ovarian tumors, PCOS, and ovarian cancers, as described by McNamara (2021) that LH/FSH ratio higher than 2 indicates PCOS or other ovarian disease. A normal range of FSH and LH/ FSH ratios was observed in healthy rats in the present research study, which proved that seed mixers with different diet ratios had no adverse effects on rats in the 4‐week study trial.

Consequently, safe levels of phytoestrogen seeds in diet during adulthood may be helpful in menarche to prevent hormonal imbalance and reduce the risk of endocrine‐based cancers (Chukwu, Oraegbunam, and Eze 2016). Similar nonsignificant (*p* > 0.05) results for testosterone were obtained in the study of Thanos et al. (2006) in adult rats fed on phytoestrogen diets of daidzein, genistein, and glycitin. However, an increasing trend was seen in both prolactin and testosterone levels with the increase in seed doses, which might be due to the potential of phytoestrogen seeds as receptor site activators to stimulate hormone production to some extent, as described in a previous study of Weber et al. (1999). Low serum fasting insulin level is a good sign of improved insulin sensitivity by seed cycling. Seeds exhibit low glycemic index properties that cause the regulation of hyperglycemia by activation of insulin receptors that ultimately lower insulin resistance, as described in previous studies of Patel and Rauf (2017): the more insulin sensitivity, the lesser its resistance and the lower the inflammation that results from metabolic syndromes. Similarly, Nestel, Cehun, and Chronopoulos (2004) found that insulin levels lowered by 3 days' regular consumption of chickpeas in one meal serving, which means phytoestrogen chickpeas helped to improve insulin sensitivity to some extent and should be part of the diet often. This might be due to the positive impact of the digestibility of fiber, which improves insulin sensitivity and glycemic control.

On the day of blood sampling, the BD, SC_2_, and SC_3_ groups were in the non‐estrous phase, while the SC_1_ group was in the estrous phase. Significantly lowest concentration of estrogen was observed in the SC_1_ group compared to other treatments, which was because of the estrous phase at sampling time (Marcondes, Bianchi, and Tanno 2002), while it was highest in the SC_3_ rat group in the non‐estrous phase (Westwood 2008). The synergic increase of progesterone with estrogen has the highest progesterone level in the SC_2_ group, as the progesterone level remains high in the non‐estrous phase. A similar trend of estrogen and progesterone to estrus cycle was described by Marcondes, Bianchi, and Tanno (2002) through histological findings in rats, while LH levels decreased significantly (*p <* 0.05) in the non‐estrus phases of SC_2_ and SC_3_ when compared with BD. Marcondes, Bianchi, and Tanno (2002) observed a short luteal phase in the pro‐estrous phase. Prolactin levels increased significantly (*p* < 0.05) in the non‐estrous phase groups, possibly due to the di‐estrus phase of BD, where it would be low. In the SC_1_ group, it increased compared to BD, as prolactin would be high in the estrous phase. Amenomori, Chen, and Meites (1970) described the same concept of prolactin levels in estrus phases. The estrogen concentration, levels of testosterone, and prolactin of all treatments were maintained in normal ranges concerning the estrus phase.

Besides phytoestrogens, the seeds used in seed cycling also contain healthy fats that significantly impact the serum lipid profile of HDL‐c, LDL‐c, TG, and TC, with liver enzymes such as ALT and AST, as presented in Table 7. Lower LDL‐c and high HDL levels strengthen the good impact of seeds. In contrast, elevated LDL‐c level leads to more oxidation of fats and oxidized LDL‐c, which, in turn, leads toward inflammation, insulin resistance, and conversion of androgens into the estrogen governed by the activity of aromatase enzymes of fat cells (Brooks et al. 2020). The high level of TG in SC_3_ might be due to the double amount of flax seeds used in the SM1 diet of SC_3_ or due to the estrogen, which tends to increase TG by activating hepatic synthesis and inhibiting the uptake of it by adipose tissues and muscles tissues due to low activity of lipoprotein lipase (Harini et al. 2015). Wu et al. (2006) found similar results for LDL‐c and TG in postmenopausal women fed sesame seeds. Our study results could be related to the study of Smink et al. (2008), who found improvement in the digestibility coefficient. This may be linked to monounsaturated and polyunsaturated fatty acids, which can form proper micelles that increase the digestive processes after easy assimilation, whereas saturated fats form irregular micelles that halt the digestive process. A drop in lipid levels in the blood may be caused by several factors, such as improved ether extract digestibility, a diet higher in fiber, improved liver function, and a decline in the liver's activity of 3‐hydroxy‐3‐methylglutaryl coenzyme A reductase, which is crucial for intestinal lipid production and absorption (Wiseman and Lessire 1987).

Meanwhile, including seeds in the diet substantially boosts the release of pancreatic enzymes and bile acids, which are responsible for breaking down fat (Umer et al. 2024; Hosseinzadeh et al. 2014). A lower concentration of liver enzymes defines good liver health. In the present study, the ALT marker was reduced toward average in SC_3_ with no improvement in AST. These results were compared with the findings of Ahmad, Akhtar, and Ali (2012), who undisclosed that ALT and AST activity increased in *Wister Albino* female rats fed on 500 mg/kg flax seeds. Both ALT and AST levels were within the normal range compared with standards for ALT (17–61 U/I) and AST (5–23 U/I). In another study of, quercetin attenuated the level of liver enzymes by decreasing lipid peroxidation in the hepatic cells. This effect was also noted in the form of an increase in the digestibility of the protein because the liver plays an essential role in protein metabolism (Zhang et al. 2006).

The histopathological examination gave significantly different results for all treatments, as shown in Figure 2a–d. Significant hormone balance due to seed cycling resulted in potential follicle growth in the ovaries. As all the study subjects were selected with a healthy status, BD showed normal histopathology of the ovarian tissues with a normal size of the antrum, granule cells, and normal thickening of theca interna and theca externa, fed with a normal BD. SC_1_ and SC_2_ gave nonsignificant (*p* > 0.05) results in ovarian tissues with depletion of cells, decreased thickness of zona placed and presence of follicular cysts, respectively. SC_3_ showed promising results with a small fluid‐filled cavity, which would be larger in the met‐estrus phase (Westwood 2008). It gave the best results among all treatments and was similar to the BD group, showing normal organ histopathology. A study on the use of flax and cinnamon extract as phytoestrogen content against PCOS also advocated the same findings that phytoestrogens help to reduce the problematic cystic follicles by reversing them and sustaining the normal physiology and histology of the ovary with their use in diet (Riaz et al. 2022).

Phytoestrogens directly interact with the synthesis rate and mechanism of their metabolism, so they can disturb the endocrinology without depending on the receptors of each hormone because these share a similar functional structure with endogenous estrogen (Wang et al. 2023). Phytoestrogens are different from other functional compounds because of their dual nature. It mainly works as antiestrogenic (0.1 nM–1 μM) when ingested in low concentrations and acts as estrogenic (> –10 μM or more) when taken in high concentrations (Almstrup et al. 2002). The dose level was different for endocrine excess immature cell production problems like PCOS, which imbalance ovarian hormone status due to abnormal expressions of ER (Mendonça et al. 2004; Xu et al. 2021). Accordingly, dose level is very important for phytoestrogens in different reproductive stages and disorders. In our study, 12 g of SM1 and 8 g of SM2 resulted in an increased trend of endogenous estrogen in the healthy rat model.

Phytoestrogens work in hormone balancing as explained that estrogen plays a role in cell specification and regulation of hormones by fine‐tuning transcriptional regulators via its three different ERα, ERβ, and G‐protein‐coupled ER‐1. Its non‐genomic actions are responsible for opening protein‐kinase cascades, while others have epigenetic mechanisms that are important regulatory entities. These include microRNAs, DNA methylation, and posttranslational histone modifications, all influenced by estrogen signaling. Phytoestrogen in the diet helps regulate the reproductive cycle and keep away ovulatory disorders by directly or indirectly on the pituitary‐ovarian axis (Vrtačnik et al. 2014). Moreover, Tamaya (2005) reported that soy supplementation exerts different impacts depending on product types in premenstrual and postmenopausal women. At the same time, daily flax seed intake affects the menstrual cycle by increasing the length of the luteal phase due to higher progesterone than estrogen in this phase.

Histopathological examination revealed more apparent corpora lutea and a significant reduction in the chance of cyst development in the therapy groups. This indicates an increased likelihood of a normal ovulation. The ovarian cortex looked healthy in the treatment groups, and the granulosa cell layer appeared normal (Bender et al. 2010). The gonadotropin‐releasing hormones (FSH and LH) thicken ovarian cells and increase corpora lutea concentration in the treatment groups. This enhancement might increase the quantity of follicles (Manni et al. 2005). Similar protective effects on ovarian health have been observed with *Lepidium sativum* seed oil, which was shown to mitigate oxidative stress and hormonal disturbances in rats (Aboul Naser, El‐Feky, and Hamed 2024).

## 5 Conclusions

Our study revealed the potential effects of seed cycling on reproductive profile, insulin, lipid, and liver enzymes in healthy rats. Higher intake of seeds showed an increase in estrogen, FSH, and LH, while a decrease in testosterone, prolactin, and progesterone. It also significantly affects LDL‐c, HDL‐c, and insulin. High‐fiber crushed seeds showed significant weight loss by suppressing appetite. Graafian follicles showed maturation with the normal size of the antrum. The right ovary's granulosa cells and theca externa also showed significant results for the SC_3_ group. No toxicity was observed, indicating that safe levels of seed cycling were practiced in the study. The above dose is for a healthy person's intake and can be introduced into the daily diet of young girls in menarche to combat hormone imbalance during menstruation. Further studies are needed to examine the receptor activity behavior of phytoestrogens upon acute versus long‐term consumption.

## Author Contributions

**Iqra Majeed:** investigation (equal), methodology (equal). **Mahr Un Nisa:** conceptualization (equal), supervision (equal), writing – original draft (equal). **Muhammad Abdul Rahim:** conceptualization (equal), supervision (equal), writing – original draft (equal). **Mohamed Fawzy Ramadan:** funding acquisition (equal), software (equal), writing – review and editing (equal). **Fahad Al‐Asmari:** funding acquisition (equal), software (equal). **Mohammed Alissa:** funding acquisition (equal), software (equal). **Eliasse Zongo:** funding acquisition (equal), software (equal).

## Ethics Statement

All the experimental protocols were approved by the ethical committee of the Government College University Faisalabad, Punjab, Pakistan.

## Consent

All authors agreed to the publication of this manuscript.

## Conflicts of Interest

The authors declare no conflicts of interest.

## Data Availability

Even though adequate data have been given in tables, all authors declare that if more data are required, the data will be provided on a request basis.
